# A Case of Early‐Onset Alzheimer's Disease in Colombia: Clinical and Genetic Insights

**DOI:** 10.1002/alz70857_104559

**Published:** 2025-12-24

**Authors:** Lina Maria Zapata‐Restrepo, Katherine L. Possin, Agustin Ibanez, Juan Carlos Rivas, Stefanie D Pina‐Escudero, Kenneth S. Kosik

**Affiliations:** ^1^ Facultad de Ciencias de la Salud, Universidad Icesi, Cali, Colombia, Cali, Colombia; ^2^ Global Brain Health Institute ‐ UCSF, San Francisco, CA, USA; ^3^ Fundacion Valle del Lili, Cali, Colombia; ^4^ Global Brain Health Institute (GBHI), University of California San Francisco (UCSF); & Trinity College Dublin, San Francisco, CA, USA; ^5^ Latin American Brain Health Institute (BrainLat), Universidad Adolfo Ibañez, Santiago, Chile; ^6^ Global Brain Health Institute (GBHI), University of California San Francisco (UCSF); & Trinity College Dublin, Dublin, Ireland; ^7^ Universidad del Valle, Cali, Colombia; ^8^ Universidad ICESI, Cali, Colombia; ^9^ Memory and Aging Center, UCSF Weill Institute for Neurosciences, University of California, San Francisco, San Francisco, CA, USA; ^10^ University of California Santa Barbara, Santa Barbara, CA, USA

## Abstract

**Background:**

Early‐onset Alzheimer's disease (EOAD) is a rare subtype of Alzheimer's, manifesting before age 65 and often linked to genetic mutations. This report describes the case of a 47‐year‐old Colombian man with EOAD and a novel pathogenic PSEN1 mutation, c.519G>T (p.Leu173Phe).

**Method:**

Case description. The patient presented with a one‐year history of progressive cognitive and behavioral decline, including memory impairment, disorientation, paranoia, and social withdrawal. A family history of EOAD in his mother and sister suggested a genetic etiology. Initial evaluations, including brain MRI and laboratory tests, were unremarkable, but neuropsychological testing and brain SPECT scan revealed significant cognitive deficits and hypoperfusion, consistent with EOAD. The patient experienced rapid progression of symptoms, including prosopagnosia, executive dysfunction, seizures, and behavioral disturbances. Despite symptomatic management with cholinesterase inhibitors, memantine, and antipsychotics, his condition deteriorated, culminating in severe dementia by age 55.

**Result:**

Whole‐genome analysis identified a novel likely pathogenic PSEN1 mutation (c.519G>T), which had not been previously reported. This variant, located in the third transmembrane domain of the PSEN1 protein, is hypothesized to disrupt γ‐secretase activity, contributing to EOAD pathogenesis. The case highlights the phenotypic variability associated with PSEN1 mutations, including cognitive and neuropsychiatric symptoms, seizures, and parkinsonism. It underscores the importance of genetic counseling and testing in suspected familial EOAD cases, particularly in populations with known genetic predispositions. Current treatments offer limited symptomatic relief, emphasizing the need for novel therapeutic approaches.

**Conclusion:**

This case of EOAD with a novel PSEN1 mutation highlights the importance of early genetic and neuropsychological evaluations for diagnosis and management. Collaborative research is essential to advance understanding and develop effective treatments for rare neurodegenerative diseases.

